# Glucose-Sensitive Biohybrid Roots for Supercapacitive Bioanodes

**DOI:** 10.1021/acsabm.4c01425

**Published:** 2024-12-03

**Authors:** Gwennaël Dufil, Julie Pham, Chiara Diacci, Yohann Daguerre, Daniele Mantione, Samia Zrig, Torgny Näsholm, Mary J. Donahue, Vasileios K. Oikonomou, Vincent Noël, Benoit Piro, Eleni Stavrinidou

**Affiliations:** † Department of Science and Technology, Laboratory of Organic Electronics, Linköping University, Bredgatan 33, Norrkoping 601 74, Sweden; ‡ Universite Paris Cite, ITODYS, CNRS UMR 7086, 15 rue J.-A. de Baïf, Paris, île-de-France F-750 13, France; § Department of Forest Genetics and Plant Physiology, Umeå Plant Science Centre, Linnéus väg 6, Umeå 901 36, Sweden; ∥ POLYMAT, University of the Basque Country, Avenida Tolosa 72, Donostia-San Sebastian 200 18, Spain; ⊥ IKERBASQUE, Basque Foundation for Science, María Díaz de Haro 3, Bilbao 480 13, Spain; # Department of Forest Ecology and Management, Umeå Plant Science Centre, Skogsmarksgränd 17, Umeå 901 87, Sweden; ∇ Department of Science and Technology, Wallenberg Wood Science Center, Linköping University, Bredgatan 33, Norrkoping 601 74, Sweden

**Keywords:** plant biohybrids, bioelectronic, conjugated polymer, energy-harvesting, glucose oxidase, enzyme immobilization, direct electron transfer

## Abstract

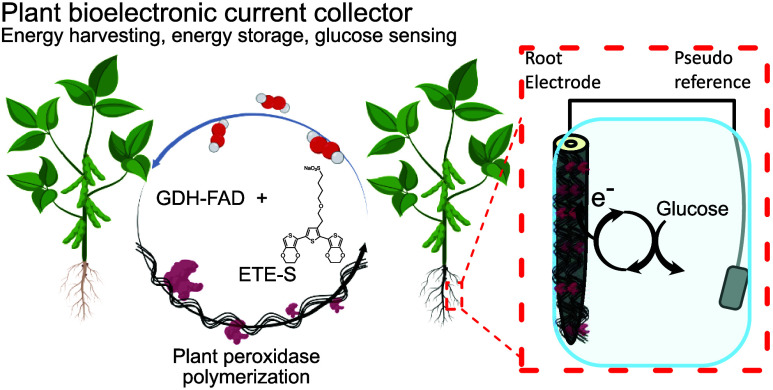

Plants as living organisms, as well as their material–structural components and physiological processes, offer promising elements for developing more sustainable technologies. Previously, we demonstrated that plants could acquire electronic functionality, as their enzymatic activity catalyzes the in vivo polymerization of water-soluble conjugated oligomers. We then leveraged plant-integrated conductors to develop biohybrid energy storage devices and circuits. Here, we extend the concept of plant biohybrids to develop plant-based energy-harvesting devices. We demonstrate plant biohybrids with modified roots that can convert common root exudates, such as glucose, to electricity. To do so, we developed a simple one-step approach to convert living roots to glucose-sensitive electrodes by dipping the root in a solution of the conjugated trimer ETE-S and the enzyme glucose dehydrogenase flavin adenine dinucleotide. The biohybrid device responds to glucose concentrations down to 100 μM while it saturates at 100 mM. The performance of our approach was compared with a classic mediator-based glucose biosensor functionalization method. While the latter method increases the stability of the sensor, it results in less sensitivity and damages the root structure. Finally, we show that glucose oxidation can be combined with the volumetric capacitance of p(ETE-S)-forming devices that generate current in the presence of glucose and store it in the same biohybrid root electrodes. The plant biohybrid devices open a pathway to biologically integrated technology that finds application in low-power devices, for example, sensors for agriculture or the environment.

## Introduction

Plants are remarkably diverse organisms that are essential for sustaining life in our ecosystem and for the prosperity of the planet. The inherent processes and structures of plants can also be leveraged for technological purposes contributing to the development of more sustainable technologies.^1^ Plants are carbon-negative as they capture more carbon dioxide than they emit, and they convert sunlight into chemical energy while releasing oxygen. Furthermore, plants have many other characteristics that were optimized via evolution such as responsiveness and acclimation to their environment, self-repair and regeneration, a plethora of biocatalytic pathways, and hierarchical structures.^2^ To enable plant-based technological systems, functional materials or devices are interfaced with plants, forming biohybrids that combine natural and artificial characteristics.

The natural processes of plants have been explored for energy-harvesting. Biofuel cells can convert naturally produced sugars and oxygen to electricity. In most examples, macroscopic electrodes prefunctionalized with enzymes and redox mediators were inserted directly into sugar-rich plant tissues such as the ground tissue of succulent plants or even fruits.^3−7^ However, these systems had a limited lifetime due to enzyme degradation and were highly invasive, as the insertion of the electrode creates a large wound in the plant tissues. Plant microbial fuel cells consist of a less invasive energy-harvesting device where electrodes are placed in the rhizosphere area, where roots release chemicals for bacterial symbiosis, to generate electricity.^8^ These devices produce enough energy to power small devices but require microbiologists to design an efficient plant/bacteria association and can be applied only with waterlogged plants.^9,10^ Apart from their chemical environment, plant movements were also leveraged for energy-harvesting. Biohybrid triboelectric generators produce electricity via the electrification phenomenon when natural and artificial leaves come in contact due to the wind.^11−15^ Artificial leaves were simply attached to the plant, rendering this approach noninvasive and these devices long lasting.

Energy storage has also been explored in plant biohybrids.^16−20^ We harnessed the plants own biocatalytic machinery to grow charge storage electrodes directly in the rich electrolytic plant tissue and fabricated supercapacitors within the plant structure.^19^ Specifically, we found that water-soluble conjugated oligomers polymerize in vivo due to the endogenous peroxidase enzymes of the plants, which are stress regulatory enzymes that tune the cell wall density through the synthesis of biopolymers.^21,22^ By distributing trimers via the vasculature or simply by watering the plants, we formed integrated conductors in the vascular tissue of plant cuttings or along the root system of intact plants. The in vivo formed charge storage electrodes had high conductivity and charge storage capacity, enabling seamlessly integrated supercapacitors in living plants.^20^

Other plant biohybrid systems aim at sensing. Most examples of plant sensor devices rely on nanomaterials that are introduced into the plant via leaf infiltration and root uptake. The nanoparticles are designed to interact with targeted analytes and to produce a readable output such as a change in fluorescence.^23^ This approach has been used to monitor endogenous biomolecules such as H_2_O_2_ or analytes that are present in the soil, for example, nitroaromatic compounds and arsenic.^24−27^ In the latter case, the plant collects and concentrates the analyte of interest into the functionalized leaf via its internal microfluidic network, the vascular tissue, which connects the root to the shoot.

Among plant organs, the root system is particularly attractive for building biohybrid systems. Roots are responsible for the uptake of nutrients and minerals required for the plant development but also release primary and secondary metabolites into the soil as exudates.^28^ These exudates have a dual function: lubricating the soil to facilitate root navigation and orchestrating the formation of the rhizosphere that includes various symbiotic microorganisms.^29^ Exudates also correlate with the physiological status of the plants and their response to various environmental stimuli. Sensors integrated in the root system of the plant can therefore provide valuable information on the dynamic processes related to plant heath and its interaction with the local environment.^30,31^

In this work, we developed biohybrid electrodes based on living plant roots that can convert glucose to current, opening possibilities for sensing and energy-harvesting applications. Leveraging the oxidative environment of the root, we fabricated in a single step a glucose-sensitive root by combining the in vivo polymerization of the conjugated trimer 4-[2-{2,5-bis(2,3-dihydrothieno[3,4-*b*][1,4]dioxin-5-yl)thiophen-3-yl}ethoxy]butane-1-sulfonate sodium salt (ETE-S) with the simultaneous immobilization of glucose dehydrogenase flavin adenine dinucleotide (GDH-FAD) in the polymer matrix. We then compared this simple approach with a classical one where glucose oxidase is immobilized in a redox hydrogel at the root-electrode surface.^32^ Both approaches led to glucose sensitivity with differences in the dynamic range, stability, and effect on the root morphology. Finally, we combined the biocatalytic current generation with the charge storage ability of the p(ETE-S) layer, demonstrating a multifunctional biohybrid root.

## Results and Discussion

**Figure 1:**
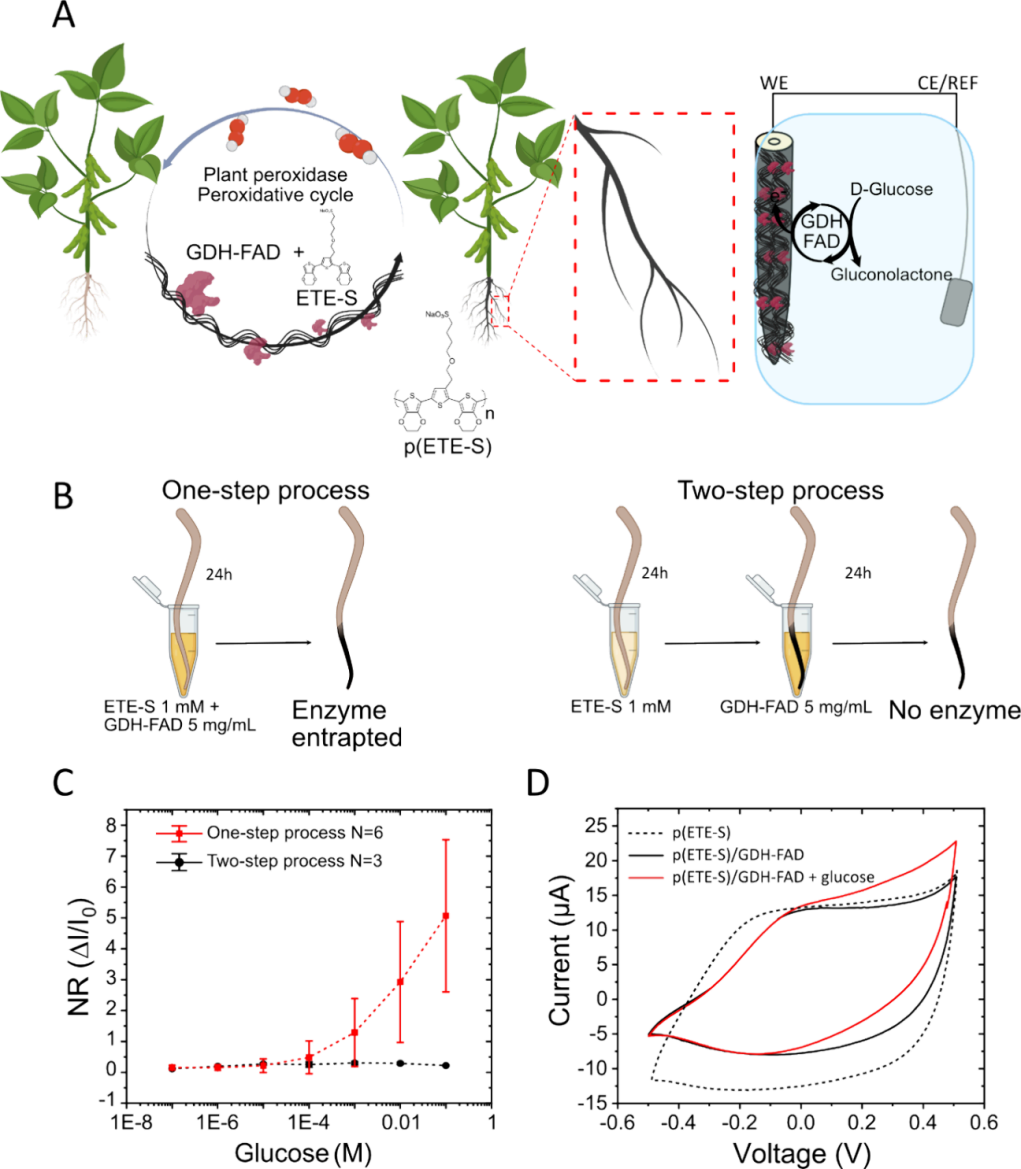
(A) Schematic representation of the root biofunctionalization method using ETE-S mixed with GDH-FAD. GDH-FAD is entrapped in the p(ETE-S) layer, while it is formed via in vivo polymerization. The biofunctionalized root then acquires glucose sensitivity. (B) The one-step process consists of polymerizing ETE-S in the same medium that contains the GDH-FAD enzyme. The two-step process consists of first polymerizing ETE-S on a root for 24 h and then transferring the root into a GDH-FAD solution for 24 h more. (C) Chronoamperometric curve for glucose detection comparing the one-step process (red, *n* = 6) with the two-step process (black, *n* = 3) for a potential of +0.5 V vs Ag/AgCl. (D) Cyclic voltammogram in 10 mM KCl at 5 mV s^–1^ vs Ag/AgCl of a p(ETE-S) root (dashed black line) and p(ETE-S)/GDH-FAD (one-step process) before (solid black line) and after the addition of 100 mM glucose (red).

The conjugated trimer ETE-S polymerizes on the root epidermis due to the presence of endogenous peroxidase enzymes forming an integrated electroactive layer with conducting and capacitive properties.^21,22^ In this study, we aimed to advance the functionality of the biohybrid roots by introducing electrocatalytic activity, specifically glucose sensitivity, via the in vivo polymerization process. We hypothesized that by having a glucose-sensing enzyme within the solution of the ETE-S monomer, the enzyme will be entrapped in the conducting matrix, while the p(ETE-S) layer is formed (Figure 1A). We selected GDH-FAD as it is one of the glucose oxidase enzymes that shows electron transfer without the need for a mediator.^33,34^ We therefore simply immersed roots of *Phaseolus vulgaris* (common bean plant) in a mixture of GDH-FAD and ETE-S for 24 h (Figure 1B).

A dark coating was formed on the root surface, indicating the in vivo polymerization of ETE-S into p(ETE-S).^21^ The roots were then mounted in a three-electrode setup for evaluating their glucose response by chronoamperometry and cyclic voltammetry (Figure 1C,D). We applied +0.5 V to the root electrode for chronoamperometry, as this potential resulted in a higher generated current without overoxidation of p(ETE-S) over time (Figure S1). The normalized response- eq 1 -showed that the root became glucose-sensitive. The absence of a soluble mediator in the system points out a direct electron transfer between GDH-FAD and p(ETE-S). Microbial GDH-FAD, like the one used in this study, has been shown to support direct electron transfer when the electrode is made of carbon nanotubes or graphene that reach very close proximity with the FAD subunit of the enzyme.^35−37^ Additionally, GDH-FAD is oxygen-independent and does not generate H_2_O_2_ as a byproduct.^38^ Therefore, in the presence of electrolyte and glucose, the current collected by the p(ETE-S) electrode can only come from the enzyme to the conjugated polymer.

Current was generated from 100 μM glucose with a linear normalized response for glucose concentrations between 1 and 100 mM. To verify that the enzyme was entrapped during the polymerization process and not just adsorbed on the polymer surface, we performed control experiments where p(ETE-S) roots were immersed in the enzyme solution after the p(ETE-S) layer formation (Figure 1B). In this case, no response to glucose was observed (Figure 1C). With cyclic voltammetry, we examined how the electrochemical properties of the roots were affected by the GDH-FAD entrapment. p(ETE-S) roots behave as capacitive electrodes in an electrochemical window delimited by the polymer overoxidation and oxygen reduction (Figure 1D).^19,39^ We observed that the capacitance is lower for the p(ETE-S)/GDH-FAD roots than for the p(ETE-S) roots possibly due to the presence of the enzyme in the conducting polymer matrix. When glucose was added to the system, an increase in current was observed for roots with enzymes. In the absence of glucose, we do not observe any redox peaks arising from the enzyme cofactor. This observation indicates that electron transfer may not occur via redox reactions. A reported work from Filipiak et al. showed a similar behavior with GDH-FAD on a single graphene sheet while Ishida et al. observed similar results with carbon nanotube electrodes.^34,36^ The latter group proposed that direct electron transfer occurs through electron tunneling, which relies on the proximity of the two redox centers, in our case p(ETE-S) and GDH-FAD.^35^ We did not investigate the mechanism that drives the enzyme entrapment and interaction with the polymer matrix in this work, but we believe that this would improve the device performance.

**Figure 2:**
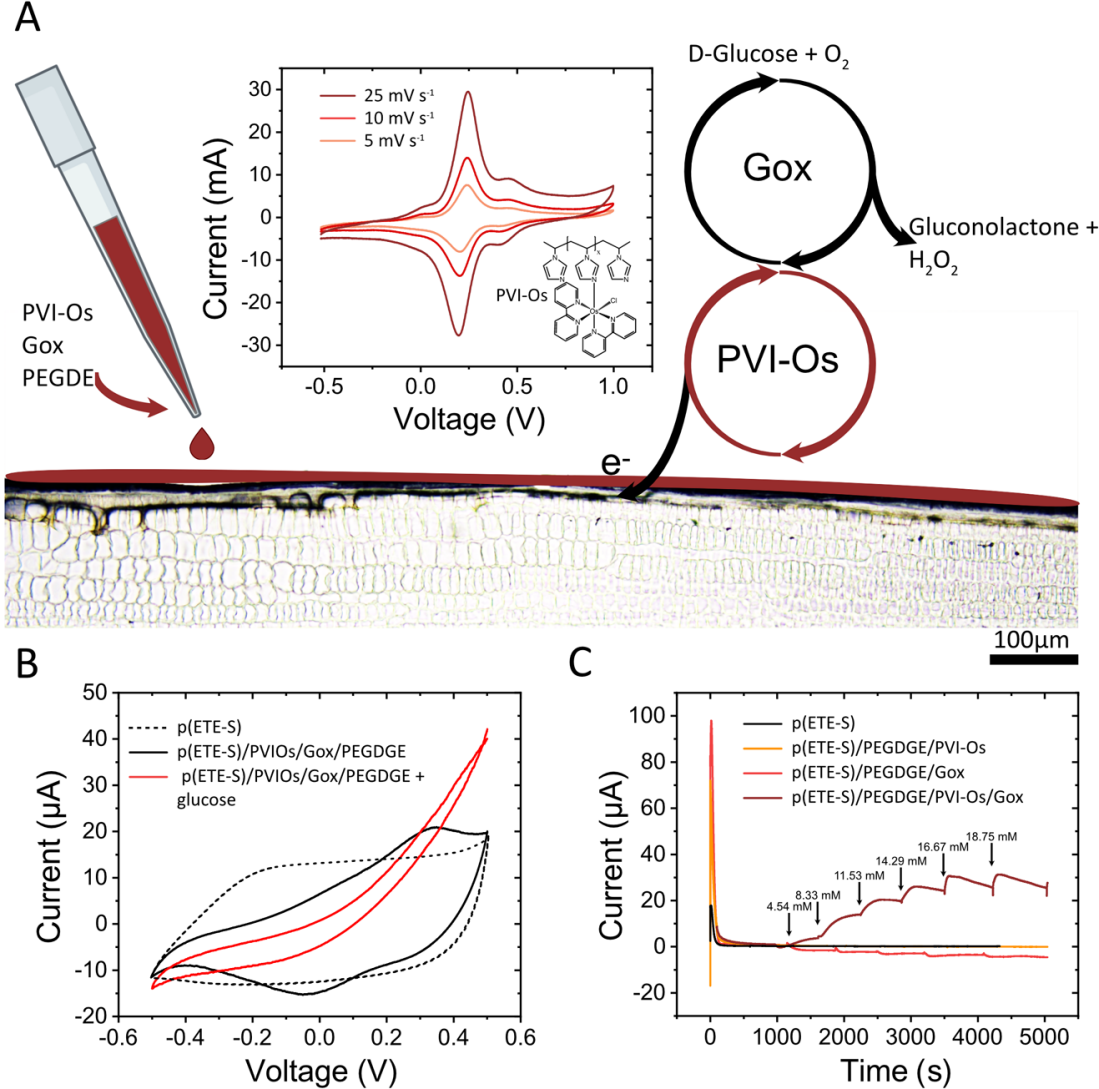
Root biofunctionalization method using a redox hydrogel matrix composed of GO*_x_*, and PVI–Os crosslinked with PEGDE and drop-cast on a p(ETE-S) root. (A) Cyclic voltammogram showing the redox activity of 1 mM PVI–Os with an electropolymerized p(ETE-S) on ITO as the working electrode in 0.1 M KCl vs Ag/AgCl at scan rates of 25 mV s^–1^ (dark red), 10 mV s^–1^ (red), and 5 mV s^–1^ (orange). Cascade reaction of glucose oxidation and transport of electrons from the redox mediator to the p(ETE-S) root. (B) Cyclic voltammogram of p(ETE-S) roots (black dotted line), p(ETE-S)/PVI-Os/PEGDGE/GO*_x_* before (black) and after the addition of 100 mM glucose (red), in 10 mM KCl electrolyte with a scan rate of 5 mV s^–1^ vs Ag/AgCl. (C) Chronoamperometry of p(ETE-S) root (black), p(ETE-S)/PVI–Os/PEGDGE root (yellow), p(ETE-S)/GO*_x_*/PEGDGE root (red), and p(ETE-S)/PVI–Os/GO/PEGDGE root (brown) with successive additions of glucose. The concentration after each addition is indicated above the arrow. The p(ETE-S) root potential is fixed at +0.5 V vs Ag/AgCl.

We then compared the one-step functionalization process with a classical mediator-based approach that is commonly used in the field of biosensors and biofuel cells.^32,40,41^ In this approach, glucose oxidase (GO_*x*_) is immobilized within a redox hydrogel: polyvinyl imidazole-osmium 2,2′-bipyridine dichloride (PVI–Os(Bpy)_2_Cl_2_) -PVI–Os in the text. In this electrochemical cascade, glucose will be oxidized by GO_*x*_, while the osmium redox hydrogel will transfer electrons from the reduced enzyme to the working electrode- the p(ETE-S) root (Figure 2A). The osmium bipyridine complexes are a well-established choice for GO*_x_* mediators as they are fast and reversible systems.^32^ In addition, osmium redox systems offer a tunable window, around 400 mV vs SCE (170 mV vs Ag/AgCl), which is in the range of the GO*_x_* oxidation potential (+100 mV vs Ag/AgCl).^42^

Initially, the electrochemical performance of the redox mediator PVI–Os was evaluated on p(ETE-S) films electropolymerized on ITO-coated glass slides. From the cyclic voltammogram, the potential of the osmium redox couple *E*_0_ (Os_2+_/Os_3+_) was found at 229 mV vs Ag/AgCl, which suggests that the redox polymer can act as a mediator for the glucose oxidase enzyme (Figure 2A). The anodic and cathodic peak currents increase linearly with the square root of the scan rate indicating that the osmium complex is able to freely diffuse to the working electrode even though it is branched on the PVI (Figure S2).^43^ Then, to test the electrochemical cascade, GO_*x*_ and glucose were added to the solution (Figure S3B), and indeed, a catalytic current was generated. As a control, we performed the same experiment without the p(ETE-S) film by having a neat ITO as the working electrode. In that case, we generated a 10 times lower current (Figure S3A). Since the mediator is in solution, the current depends on the diffusion layer around the electrode surface; the p(ETE-S) film increases the surface area of the electrode and therefore the interaction with the mediator.

To develop the biohybrid glucose-sensitive electrodes, we first functionalized the roots of young bean plants with p(ETE-S) to form an integrated conductor and biofunctionalized the conducting roots by casting a matrix containing GO_*x*_ and PVI–Os crosslinked with poly(ethylene glycol) diglycidyl ether (PEGDGE). The epoxy rings of PEGDGE covalently bind the amine groups at the surface of the enzyme.^32,44^ The roots were then mounted in a three-electrode setup for evaluating their glucose sensitivity using cyclic voltammetry and chronoamperometry.

Figure 2B shows the cyclic voltammogram of the p(ETE-S) roots with and without the addition of a glucose-sensing matrix. The biofunctionalized roots were less capacitive than the p(ETE-S) roots. The black solid line peaks at 347 and −41 mV- respectively in the anodic and cathodic currents- signifying that the redox hydrogel can be addressed with the p(ETE-S) root electrode. The peak-to-peak difference (Δ*E*_p_), however, was larger than in the case where PVI–Os was in solution, signifying that a higher potential must be applied for the redox reaction to take place; There is a higher resistivity for the electron transferwith the p(ETE-S)-ITO electrode.

When we added glucose to the solution, we observed a high catalytic activity resulting in high current generation. At the same time, the volumetric capacitance of p(ETE-S) was lost, with a visible decrease in the cyclic voltammogram width in the noncatalytic region. This shows that the modified p(ETE-S) root during glucose oxidation no longer acts as a volumetric capacitor but only as a resistor. The capacitance of the electrode recovers slowly during cycling, while the catalytic current decreases (Figure S4).

To assess the response of the biofunctionalized root to glucose, we performed chronoamperometry in solution with an increasing amount of glucose. We observed a linear increase in the current for glucose concentrations ranging from 4 to 14 mM (Figure 2C and Figure S5). To verify that all components of the biofunctionalization matrix were taking part in the reaction, we performed a series of sensing experiments for roots modified with PEGDGE/PVI–Os, PEGDGE/GO*_x_*, and PVI–Os/GO*_x_*/PEGDGE. No current increase was observed upon glucose addition for nonmodified p(ETE-S) roots- in the absence of GO*_x_*- while when p(ETE-S) roots were modified with GO*_x_* crosslinked with PEGDGE, we observed a reduction current 10 times lower than the one generated by the oxidation of glucose with p(ETE-S)/PVI–Os/GO*_x_*/PEGDGE (Figure 2C). It has been shown that conducting polymers such as PEDOT can favor the reduction of oxygen into hydrogen peroxide but not in potential windows in which we were carrying out the experiments.^45^ More experiments should be carried out to elucidate the source of the reduction current.

**Figure 3:**
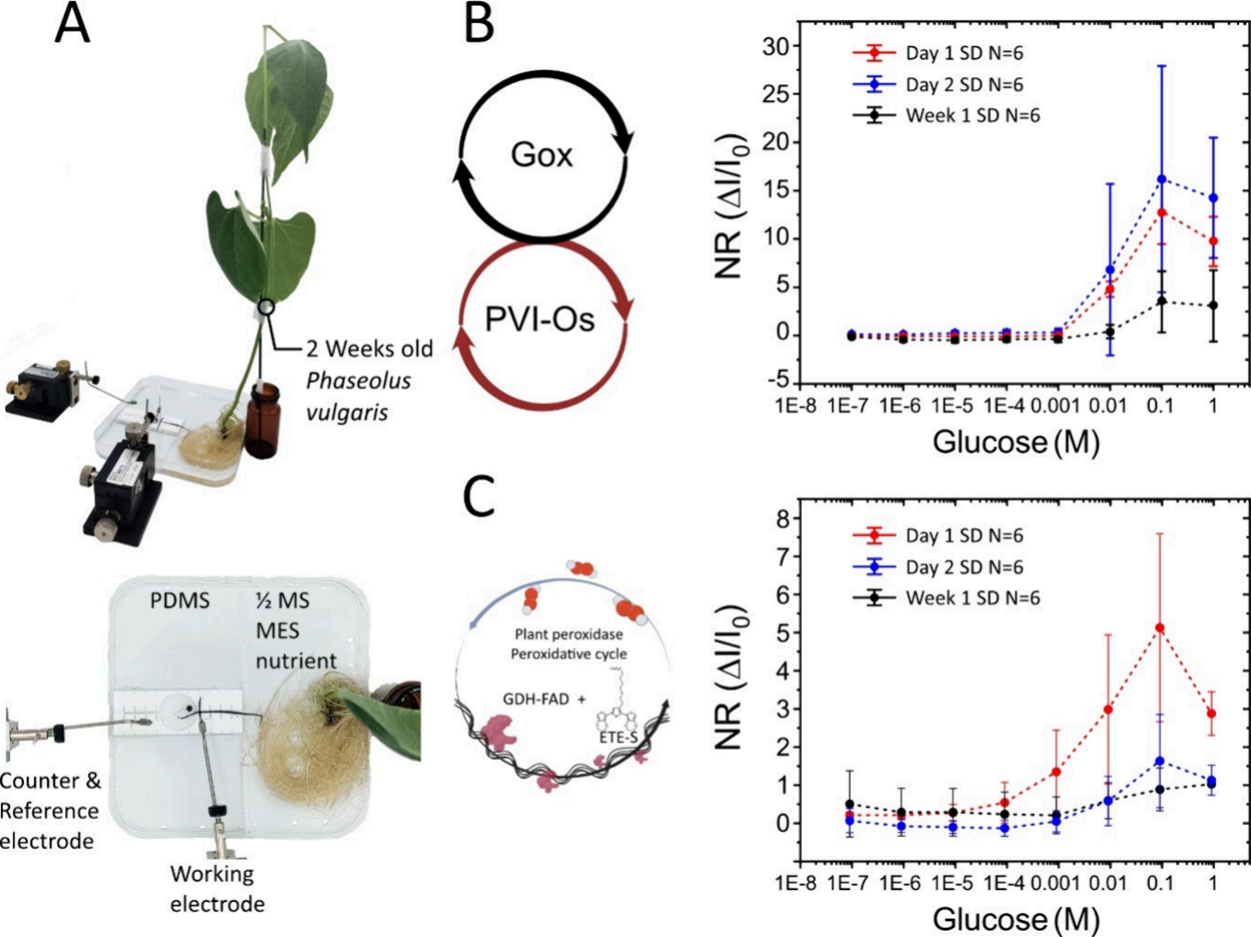
Glucose sensing in intact plants. (A) Image of the sensing measurement setup with an intact plant. A 2-week-old plant is placed in a 1/2 MS MES nutrient medium without sugar, while the biofunctionalized root (still attached on the plant) is placed in an electrolytic cell with 10 mM KCl. (B) Normalized response of glucose sensing measurement of a p(ETE-S)/PVI–Os/GO_*x*_/PEGDGE root at +0.5 V vs Ag/AgCl. Measurement on day 1 (red), day 2 (blue), and 1 week after biofunctionalization (black). (C) Normalized response of glucose sensing measurement of a p(ETE-S)/GDH-FAD root at +0.5 V vs Ag/AgCl. Measurement on day 1 (red), day 2 (blue), and 1 week after biofunctionalization (black). Standard deviation obtained for *N* = 6 replicates.

Next, we investigated the performance and stability of p(ETE-S)/PVI–Os/GO*_x_*/PEGDGE and p(ETE-S)/GDH-FAD roots while still being attached to the plant (Figure 3A). To do so, we developed a setup where the biofunctionalized root is placed in an electrochemical cell with 10 mM KCl while the rest of the root system is placed in a sugar-free 1/2 MS MES medium at pH 5.8. The glucose response of the functionalized roots was assessed on the day of the biofunctionalization, after 1 day, and after 1 week. Plants were placed back in the hydroponics growth chamber after each measurement. The biohybrid root was biased at +0.5 V vs Ag/AgCl, and- after the current stabilized- the glucose concentration of the solution was increased stepwise from 100 nM to 1M. In the case of the p(ETE-S)/PVI–Os/GO_*x*_/PEGDGE root, we observed a glucose response at 1 mM, saturation at 100 mM, and a decrease at 1 M glucose concentration (Figure 3B). Sensing curves corresponding to the day of functionalization and 1 day after showed similar responses. However, after a week, a decrease in the performance was observed, indicating that the activity of the enzyme was reduced.

Figure 3C shows that roots with p(ETE-S)/GDH-FAD produce a 4 times lower normalized response than the p(ETE-S)/PVI–Os/GO*_x_*/PEGDGE roots. We speculate that in the first case the electron transfer to the p(ETE-S) electrode is limited as it relies on the physical distance between the enzyme cofactor and the conducting layer. For the p(ETE-S)/PVI–Os/GO*_x_*/PEGDGE roots, on the other hand, the redox mediator facilitates the transfer from the enzyme cofactor to the electrode. However, p(ETE-S)/GDH-FAD roots showed a response to glucose starting at 100 μM concentration but decreasing 1 day after the functionalization (Figure 1C). This indicates that the enzyme is either no longer active or present in the vicinity of the p(ETE-S) network of the root. The literature shows that glucose dehydrogenase activity notably decreases under continuous operation at room temperature.^46,47^ Despite the difference in performance and stability, SEM micrographs (Figure S6) revealed that in p(ETE-S)/PVI–Os/GO*_x_*/PEGDGE roots, the crosslinked matrix altered the root morphology. In contrast, in p(ETE-S)/GDH-FAD, the root structure was preserved. Maintaining the root morphology and function is vital for nutrient uptake and exudation. Root exudation is particularly relevant, as it involves the release of glucose and other sugars in the soil that could be directly oxidized by the biohybrid roots. To evaluate the sugar exudation of the bean plants, we collected exudates from adult plants and quantified their glucose and sucrose concentration using gas chromatography coupled with mass spectrometry (GC–MS; Methods in the Supporting Information). We found that sucrose exudation was 1 order of magnitude higher than that of glucose with respective values of 34.88 and 1.62 nM h^–1^ per plant (Figure S7). No significant difference in the exudation was found between the control and ETE-S root systems, indicating that ETE-S functionalization does not negatively affect the root exudation of glucose and sucrose, possibly since glucose and sucrose have no electric charge. The biohybrid roots generate notable current when the glucose concentration is higher than 100 μM. This concentration can be reached in 17 min for sucrose and 370 min (6 h) for glucose in 100 μL of growth medium. Therefore, the biohybrid roots can be used for sensing or energy-harvesting using a miniaturized compartment or by enhancing the glucose sensitivity.

**Figure 4:**
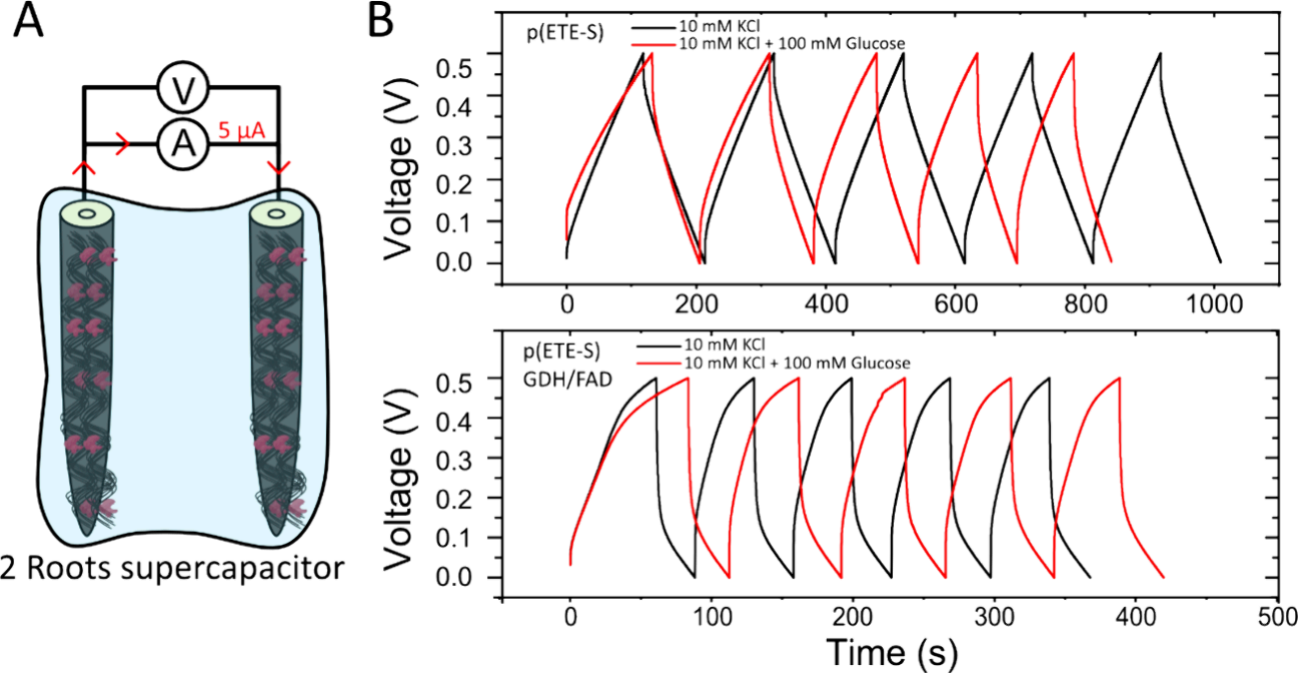
ETE-S/GDH-FAD roots are supercapacitive bioanodes. (A) Schematic representation of the experimental setup for the galvanostatic charge/discharge characterization of two roots functionalized with 1 mM ETE-S + 5 mg mL^–1^ GDH-FAD. (B) Galvanostatic charge/discharge curve for a ±5 μA applied current for a voltage range of [0, +0.5] V. The electrolyte used here is either 10 mM KCl (black) or 10 mM KCl electrolyte +100 mM glucose (red) for 2 roots functionalized with ETE-S (upper graph) and 2 roots functionalized with ETE-S + GDH-FAD (lower graph).

So far, we have established that roots modified with glucose oxidase enzymes can catalyze the oxidation of glucose. As the next step, we aimed to combine the biocatalytic current generation with the charge storage ability of the p(ETE-S) layer on the roots. To demonstrate the dual function of the enzyme-modified roots, we performed galvanostatic charge–discharge characterization of a pair of roots in a supercapacitor configuration with or without glucose present in the electrolyte (Figure 4A). We characterized the response of a supercapacitor made with roots modified only with p(ETE-S). As we have shown previously, p(ETE-S) is a mixed ionic/electronic conductor,^19^ and p(ETE-S) roots show capacitive charging without the generation of a faradic current. We calculated the capacitance from the linear discharge curves- area with a constant capacitance-for -5 μA applied, -. and the equivalent series resistance (ESR) from the ohmic drop of the discharge curve.

The capacitance of the p(ETE-S) supercapacitor was 1.2 mF and decreased to 1.1 mF with the addition of glucose. p(ETE-S)/GDH-FAD roots exhibited lower capacitance values of 1 and 0.80 mF before and after glucose addition, respectively (Figure S8). This correlates with the conductivity recorded with the 4-point probe measurement of p(ETE-S) roots and p(ETE-S)/GDH-FAD roots (49 ± 31 and 2 ± 1 S cm^–1^, respectively; Figure S9), as the conductivity and capacitance depend on the coupling between electronic and ionic carriers within the conjugated polymer matrix.^48,49^

p(ETE-S)/GDH-FAD roots indicate a larger ESR (34 kΩ) than the p(ETE-S) root supercapacitors (6 kΩ), which confirms the lower conductivity of our modified root; it is harder for the electronic charges to cross the polymer with the embedded enzyme present in the structure.

When we added glucose in the electrolyte, we observed different charging responses with a plateau developing when the voltage became higher than the oxidation potential of the GDH-FAD enzyme. Overall, we can conclude that the biofunctionalized roots demonstrate a dual function that combines both current generation via glucose oxidation and the charge storage capacity with the p(ETE-S) mixed conducting layer.

## Conclusions

In this work, we demonstrate an elegant method to produce in one step an electronic root that converts glucose to current by simply placing a root in a solution with the conjugated trimer ETE-S and the glucose dehydrogenase enzyme (GDH-FAD). The endogenous peroxidase enzymes on the root epidermis catalyze the polymerization of ETE-S to a conducting layer that, at the same time, entraps the enzyme. We show clear glucose oxidation catalyzed by the enzyme and the collection of the electronic charge via the self-organized p(ETE-S) layer on the root epidermis. We then compared the performance of p(ETE-S)/GDH-FAD with p(ETE-S) roots that were functionalized with a redox mediator hydrogel branched with glucose oxidase with PEGDE (PVI–Os/GO*_x_*/PEGDGE/p(ETE-S) roots). We found that while in the first case the sensitivity increased by 1 order of magnitude, overall lower currents were generated due to the absence of a mediator and the dependence on direct electron transfer from the enzyme to the electrode. GDH-FAD was also less stable than GO*_x_* as we observed a clear drop in sensitivity after 1 day. On the other hand, the PVI–Os/GO*_x_*/PEGDGE/p(ETE-S) root morphology was greatly impacted due to the use of a crosslinker in the biofunctionalization process . Osmium complex degradation in nature also poses inherent risks due to the toxicity of osmium oxides.^50^ These facts could hinder the implementation of such devices in living plants. Furthermore, we quantified the sugar exudation of our plant model and found that the biohybrid roots can, in principle, be used to sense metabolites from living plants. However, further development would be needed to enhance the sensitivity and stability of the biohybrid roots. Finally, we combined the glucose oxidation catalytic matrix with the inherent volumetric capacitance of the p(ETE-S). We demonstrated that the root p(ETE-S)/GDH-FAD could dually extract the current from a glucose source and store the charge at the conjugated polymer bulk. Such devices could potentially find application in biofuel cells for powering low-energy consumption devices such as environmental sensors or local delivery systems.^51^

## Methods

### Cyclic Voltammetry of 1 mM PVI–Os(Bpy)_2_Cl_2_ on ETE-S

An ITO-coated glass (Ossila) was placed in an electrochemical cell (BMM EC 15 ML, Redox.me) and used as the working electrode with a Pt wire counter electrode and 3 M KCl-saturated Ag/AgCl as the reference electrode. For the electropolymerization of ETE-S, a 1 mM ETE-S solution in 0.1 M KCl was used and a chronoamperometric deposition was performed (+0.4 V vs Ag/AgCl) for 500 s. After deposition, the cell was rinsed several times and the electrolyte was replaced by 1 mM *PVI–Os(Bpy)*_*2*_*Cl*_*2*_ (*M*_w_ = 1150.9 g mol^–1^) diluted in 0.1 M KCl. The solution was purged for 15 min using N_2_ gas prior measurements.

### Plant Growth

*Phaseolus vulgaris* seeds (Impecta Fröhandel) were germinated in peat-based cubes for 5 days (Root riot) in the dark at 23 °C. After germination, plants were transferred to a hydroponic chamber filled with 0.2% vol/tap water commercial nutrient solution (Nelson Garden). Hydroponics chambers were placed for 9 days in a controlled growth greenhouse environment with the following conditions: 23–25 °C, humidity 50–60%, and 12h light/dark cycles with neon lights providing a light intensity of 80–100 mmol m^–2^ s^–1^.

### Root Biofunctionalization with Glucose Oxidase, *PVI–Os(Bpy)*_*2*_*Cl*_*2*_, and PEGDGE

One root of the plant was isolated and placed in 0.5 mL of a solution of 1 mM ETE-S overnight. The next day, the root was removed, washed several times with DI water, and placed on a parafilm foil with a 20 μL drop of the biofunctionalization medium following the protocol from Taylor et al.^32^ and allowed to dry overnight. The biofunctionalization medium consists of 5 μL of 5.6 mg mL^–1^ PEGDGE (Mn= 500 Da, Sigma-Aldrich), 5 μL of 16 mg mL^–1^ glucose oxidase from *Aspergillus niger* (Type X–S 100–250 kU g^–1^ solid, Sigma-Aldrich), and 10 μL of 20 mg mL^–1^*PVI–Os(Bpy)*_*2*_*Cl*_*2*_ (*M*_w_ = 1150.9 g mol^–1^, Paris Diderot University). The next day, the root was washed several times and used directly for the glucose-sensing experiments.

### Root Biofunctionalization with GDH-FAD

One root was isolated and placed overnight in 0.5 mL of a solution containing 1 mM ETE-S and 5 mg mL^–1^ GDH-FAD (500 U mg^–1^, GLD- 3, BBI solutions). The next day, the root was washed 5 times with DI water before being used for the glucose-sensing experiments.

### Biohybrid Root Electrical Contact

We achieved contact with the root using micropositioners with a Au-plated tungsten probe (50 μm tip) from Quarter Research (model XYZ300). Roots were placed on a glass Petri dish with perpendicularly aligned carbon fibers as contact electrodes for the roots. Tungsten probes coated with carbon paste, which improve the smoothness of the tip, were gently positioned on top of the carbon fiber to electrically address them. The probe was brought closer to the carbon fiber with a stereomicroscope to ensure soft contact and avoid going through the root sample.

### Glucose Sensing Setup

The 3D printed electrolytic cell (PLA, AA 0.4, scheme available in SI) was placed in a square Petri dish filled with polydimethylsiloxane (PDMS, Sylgard 184, Dow Corning, 10:1) and cured overnight at 50 °C. Kapton tape was used to delimit the PDMS areas. The day after, the plant was attached to a stand and immersed in a 1/2 MS MES medium on the half of the Petri dish without PDMS. The counter/reference electrode was an Ag/AgCl electrode (2.0 × 4 mm, World Precision Instruments). The 1/2 MS MES was prepared from reagents purchased from Sigma-Aldrich. A micronutrient stock of 1 L was prepared with: 4.460 g of MnSO_4_·4H_2_O, 1.720 g of ZnSO_4_·7H_2_O, 1.240 g of H_3_BO_3_, 166 mg of KI, 50 mg of NaMoO_4_·2H_2_O, 5 mg of CoCl_2_·6H_2_O, and 5 mg of CuSO_4_·5H_2_O. A micronutrient stock solution of 1 L was prepared with: 367.08 mg of FeNaEDTA, 3.695 g of MgSO_4_·7H_2_O, and 1.7 g of KH_2_PO_4_. A 1 L stock solution of CaCl_2_ was prepared with 4.395 g of CaCl_2_·2H_2_O. A 1 L stock solution of KCl was prepared with 7.456g of KCl. To prepare 1 L of 1/2 MS MES, we added:2.5 mL of micronutrient stock, 50 mL of macronutrient stock, 50 mL of CaCl_2_ solution, 1.5 g of MES, 303 mg of KNO_3_, and 7 mL of KCl solution. The pH was adjusted to 5.8 before we completed the volume of 1L with Milli-Q water and sterilized the bottle in an autoclave.

### Glucose Sensing

To obtain Figure 2C and Figure S5, the glucose sensing was performed as follows: sensing experiments were conducted in a 1 mL drop of 10 mM KCl with the addition of 100 μL of 50 mM d-glucose every 10 min in a 3-electrode setup. After 30 min, a 100 μL drop of d-glucose in 10 mM KCl was added to the initial drop every 10 min. To obtain Figure 3B and C, a serial dilution of d-glucose was performed from a 1 M stock solution to attain a concentration of 100 nM. After placing the root in the glucose sensing setup, the glucose concentration was increased by switching the previous concentration to the next one. Data were acquired using a source meter Keithley (K2600B) with a potential of +0.5 V vs Ag/AgCl applied to the functionalized root and a data recording frequency of 10 Hz. The normalized response was determined using the formula:
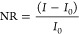
1

### Collection of Sucrose and Glucose Exudate for GC–MS

Plants (*Phaseolus vulgaris*) were germinated for 4 days before being transferred to a hydroponic system in 1/2 MS MES medium (pH 5.8) + 3 mM KNO_3_. 20 days after germination, plants are placed in 15 mL of 1 mM ETE-S in 1/2 MS MES medium (pH 5.8) + KNO_3_ with or without 1 mM ETE-S. Plants were rinsed 24 h later in three consecutives baths of 300 mL of 0.1 mM CaCl_2_ and then transferred to 20 mL of 0.5 mM CaCl_2_ to collect the exudate for 24 h. 9 mL was kept in the vials, freeze-dried, and redissolved in 1 mL of DI water for GC–MS.

### Galvanostatic Charge–Discharge Curve

Two roots treated with ETE-S or two roots treated with ETE-S/GDH-FAD were placed in parallel. A galvanostatic program created on Lab View was used to control a Keithley source meter (K2600B). A constant current of 5 μA was applied, and the current was then reversed when the accumulated potential reached +0.5 V. Charge and discharge curves were obtained in a 10 mM KCl electrolyte with and without the presence of 100 mM glucose. The capacitance of the root was then extracted from the linear regime of the discharge curve using the following formula:
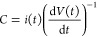
2where *I* is the applied current, and d*V*/d*t* is the slope obtained from a linear fit of the discharge curve.

The ESR was also extracted from the galvanostatic charge–discharge curve using the following formula:
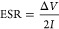
3where *I* is the applied current and Δ*V* is the voltage difference measured at the drop of the discharge curve behavior.

### Multielectrode Array Fabrication

A conformable multielectrode array (MEA) was fabricated to interface with the biohybrid roots. The array consists of 8 electrodes with dimensions of 450 μm × 200 μm in a 2 × 4 arrangement. The device was fabricated with microfabrication, as described previously.^52^ Briefly, the substrate is a 2 μm thick, flexible parylene-C layer that is deposited by chemical vapor deposition (Diener electronic GmbH) on clean glass microscope slides. The glass was cleaned by ultrasonication first in 2% Hellmanex in DI water, followed by acetone and then isopropanol. The conductive interconnects were patterned using a lift-off process with a negative photoresist (AZ nLof 2070), an MA6 Suss mask aligner with an i-line filter, and a developer (AZ 326 MIF). Following patterning of the photoresist, a 5 nm titanium adhesion layer and 80 nm thick gold layer were thermally evaporated onto the substrates, and lift-off was carried out in acetone. The metal interconnects provided the electrode contacts and allowed electrical connection to the back-end contact pads for wiring upon completion. Next, an O_2_ plasma process was carried out (2 min, 50 W), and a second 1.5 μm thick insulating parylene-C layer was deposited over the metal lines (using an adhesion promoter in the deposition chamber, A-174). The outline shape of the probes was defined using reactive ion etching (RIE, O2/CF4 gases, 150 W) after patterning with a photoresist etch mask (AZ 10XT). The etch mask was removed with an acetone wash followed by an isopropanol rinse. Afterward, the electrode surface and back contact pads were opened using the same RIE etch process with the AZ 10XT photoresist etch mask to define and provide a back-end contact possibility. The etch mask was removed from the substrate using acetone and isopropanol, and finally, the completed probes were removed from the glass substrates using DI to assist the process.

### Electropolymerization of ETE-S on Gold to Obtain the Gold Microarray

A MEA was fitted on a Kapton film to rigidify the contact area of the electrode.. Commercial nail polish was applied between the electrodes and contacts- front and back side of the array- to prevent electrolyte leakage to the contacts of the MEA. Next, the MEA was treated with UV/O_3_ for 2.5 min- 50 W power output and an O_2_ pressure of 0.6 bar (Plasma cleaner Zepto-W6). The polymerization of ETE-S was achieved using cyclic voltammetry between [−0.5 and +0.5]V vs Ag/AgCl for 8 cycles with a drop of 1 mM ETE-S in 10 mM KCl electrolyte.

### 4-Point Probe Measurement

The MEA was placed on the roots functionalized with p(ETE-S) and p(ETE-S)/GDH-FAD. A current *I* between 1 and 10 μA was then delivered between contacts 2 and 8 of the array using a source meter Keithley (K2600B) apparatus. The voltage drop Δ*V* between the two inner resistances was then recorded and used to calculate the sheet resistance with the following formula:
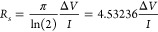
4

## Supporting Information Available

Cyclic voltammetry of p(ETE-S) on ITO substrates, reduction/oxidation peaks of Os^2+^/Os^3+^ versus (v)^1/2^, cyclic voltammetry of PVI–Os catalysis on ITO with/without p(ETE-S), cyclic voltammetry of p(ETE-S) capacitance recovery after catalysis, normalized response of p(ETE-S)/PEGDE/Go*_x_*/PVI–Os with the addition of glucose, SEM image of the root morphology after enzyme deposition, metabolomics of root exudates, extracted data of charge/discharge curves, and precision of the 4-point probe measurements (PDF)
