# Supplementary material for: Glucose-Sensitive Biohybrid Roots for Supercapacitive Bioanodes
Source: ACS Appl Bio Mater. 2024 Dec 3;7(12):8632–41. doi: 10.1021/acsabm.4c01425 (PMC11653237; doi:10.1021/acsabm.4c01425)
Supplement: Supplementary file 1 — mt4c01425_si_001.pdf [file mt4c01425_si_001.pdf]

Supporting Information

Glucose sensitive biohybrid roots for supercapacitive bioanodes

Gwennaël Dufil,[1*] Julie Pham,[2] Chiara Diacci,[1] Yohann Daguerre,[3] Daniele Mantione,[4,5] Samia Zrig,[2] Torgny Näsholm,[6] Mary J. Donahue,[1] Vasileios K. Oikonomou,[1,7] Vincent Noel,[2] Benoit Piro,[2] and Eleni Stavrinidou.[1,3,7*]

1: Linköping University Department of Science and Technology, Laboratory of Organic Electronics, Bredgatan 33, Norrkoping, SE 601 74

2: Universite Paris Cite, ITODYS, CNRS UMR 7086, 15 rue J.-A. de Baïf, Paris, Île-de-France, FR F-750 13

3: Umeå Plant Science Centre, Department of Forest Genetics and Plant Physiology, Linnéus väg 6, Umeå, SE 901 36

4: University of the Basque Country, POLYMAT, Avenida Tolosa 72, Donostia-San Sebastian, ES 200 18

5: Basque Foundation for Science, IKERBASQUE, María Díaz de Haro 3, Bilbao, ES 480 13

6: Umeå Plant Science Centre, Department of Forest Ecology and Management, Skogsmarksgränd 17, Umeå, SE 901 87

7: Linköping University Department of Science and Technology, Wallenberg Wood Science Center, Bredgatan 33, Norrkoping, SE 601 74

*corresponding authors: gwennael.dufil@liu.se, eleni.stavrinidou@liu.se

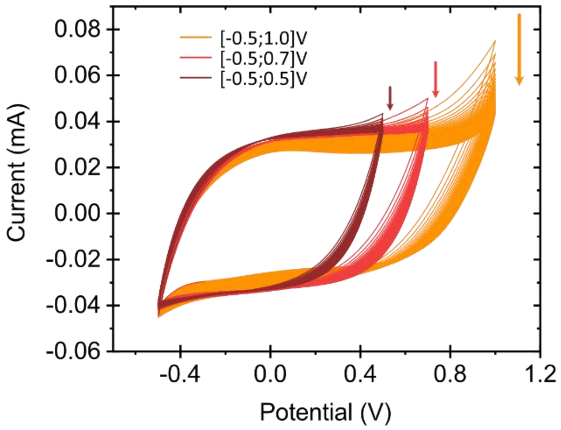

Figure S1: Cyclic voltammogram of an ITO/p(ETE-S) in 10 mM KCl at 25 mVs^{-1}, between [-0.5;+1]V (yellow), between [-0.5;+0.7]V (orange), and between [-0.5;+0.5]V (red). Ag/AgCl (3M KCl sat.) is used as a reference electrode and a Pt wire as counter electrode.

Cyclic voltammogram of an ITO/p(ETE-S) electropolymerized with chronoamperometric (CA) methods for 500s at +0.4V vs Ag/AgCl. The stability of the p(ETE-S) is tested over time for different potential windows to establish applied potential limits of our system. We observe that p(ETE-S) starts overoxidizing at 0.7V vs Ag/AgCl. CV at higher voltages causes loss of electroactivity as shown with a narrowing of the box shape.

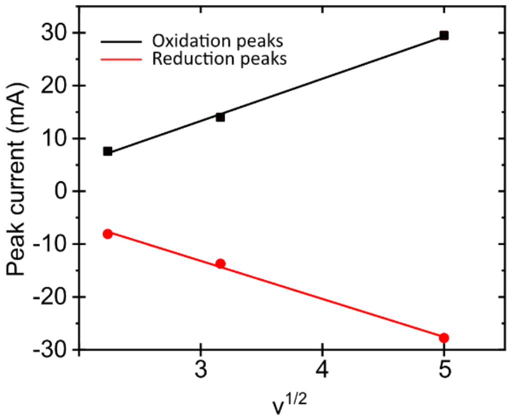

Figure S2: Maximum peaks for the Os^{2+}/Os^{3+} redox couple extracted from a cyclic voltammogram of PVI-Os with scan rates of 5, 10, and 25 mV s^{-1}.

Fitting reveals that the oxidation and reduction peaks currents change linearly with the square root of the scan rate in accordance with the Randles–Sevcik equation. R^2=0.998 for oxidation and R^2=0.997 for reduction.

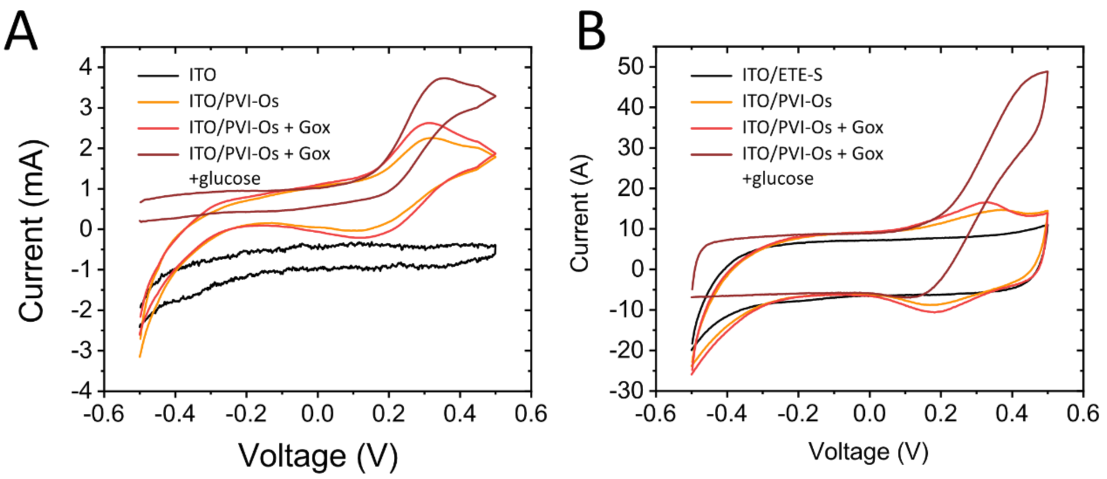

Figure S3: Cyclic voltammogram of PVI-Os in solution with GOx and glucose. A) Response of bare ITO electrode (black), with 1mM PVI-Os in solution (yellow), 1mM PVI-Os + 7 KU of Gox (orange), and 1 mM PVI-Os + 7 KU GOx and 100 mM Glucose (red). B) Response of ITO/ETE-S (black), with 1mM PVI-Os in solution (yellow), 1mM PVI-Os + 7 KU of GOx, and 1 mM PVI-Os + 7 KU GOx and 100 mM Glucose. In A and B 10 mM KCl is used as a supporting electrolyte, scan rate of 10mV s^{-1}, Ag/AgCl (3M KCl sat.) as a reference electrode, Pt as a counter electrode. ITO/ETE-S electrodes were obtained after CA deposition of 1 mM ETE-S in 10 mM KCl for 500s at 0.4V.

This data supports that the electrochemical cascade involving PVI-Os(Bpy)$_2$Cl$_2$ (PVI-Os) and glucose oxidase occurs in presence of glucose in solution. We observed that with electropolymerized p(ETE-S), the amplitude of current collected from glucose is increased by 10 times, showing that p(ETE-S) contributes fully as a current collector that catalyzes glucose oxidation.

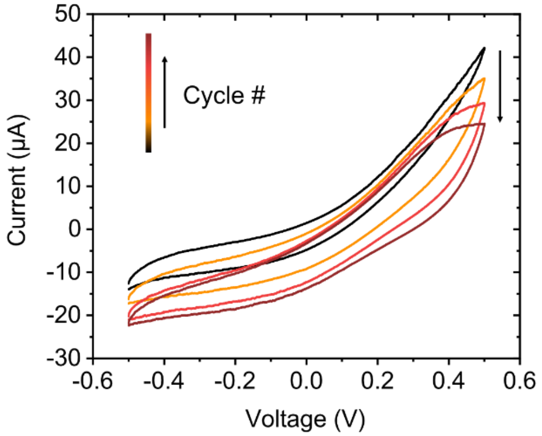

Figure S4: Cyclic voltammogram of a p(ETE-S)/PVI-Os root in 10 mM KCl, and 100 mM Glucose at 5mV s^{-1} scan rate. Increasing cycles are indicated with the black arrow. The p(ETE-S)/PVI-Os root is acting as a working electrode and a Ag/AgCl pellet as a counter-reference electrode.

This Figure shows that the increase of cycle number is associated with a broadening of the cyclic voltammogram characteristic to the volumetric capacitance of p(ETE-S).

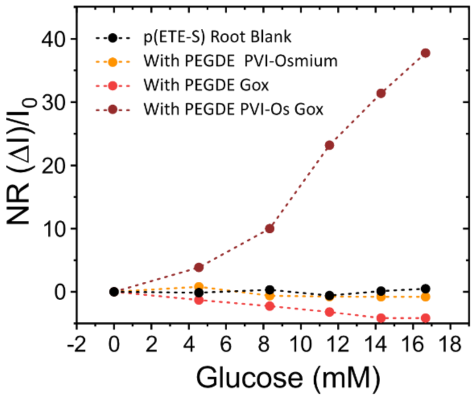

Figure S5: Normalized response of treated roots under addition of glucose. Root treated with p(ETE-S) (black). p(ETE-S) root modified with PEGDE, PVI-Os (yellow), with a PEGDE, GOx (red), and with PEGDE, GOx, and PVI-Os (brown).

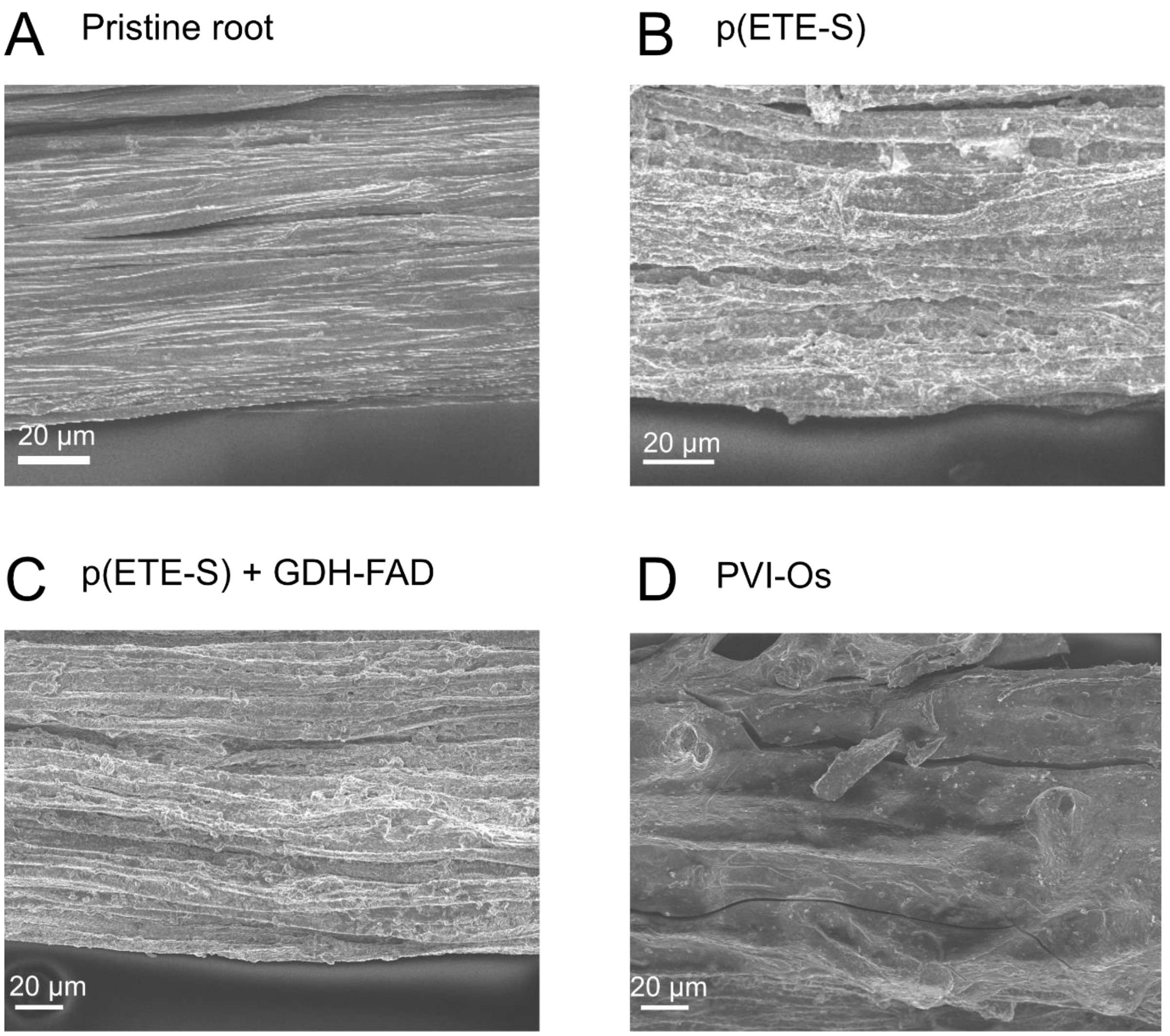

Figure S6: SEM micrographs of biohybrid roots. A) Control root. B) p(ETE-S) root. C) p(ETE-S)/GDH-FAD root. D) p(ETE-S) + PVI/GOx/PEGDGE root.

The SEM micrographs reveal the microscopic morphology of the roots for the different treatments. From figure A, we can observe the cellular structure of the root. In figure B, we can observe that the addition of the p(ETE-S) layer gives a rough aspect with grains that corresponds to the the polymer over the plant cell wall. The blend of p(ETE-S) and GDH-FAD (Figure C) shows similar features with the presence of less grains. On the other hand, we can observe that the coating of PVI-Os/GOx/PEGDGE changes the entire structure of the root eliminating the fiber features at the surface. This supports the loss of mechanical integrity of the root that we have observed with those sensors.

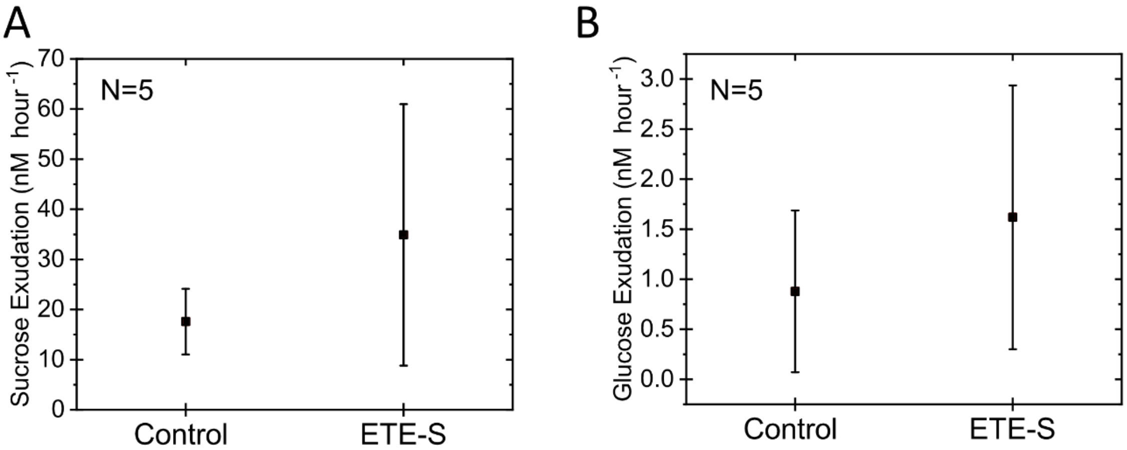

Figure S7: Concentration of sucrose (A) and glucose (B) in plant exudates for plant treated or untreated with ETE-S. SD for N=5

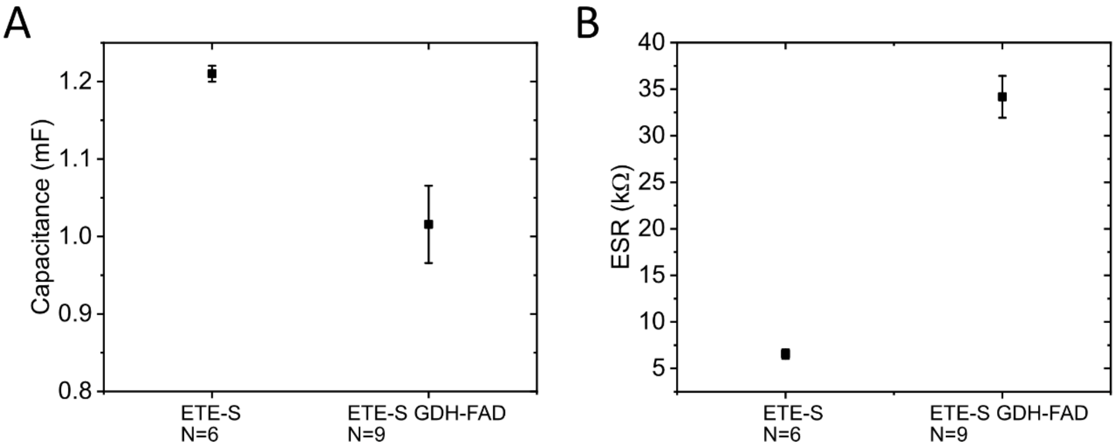

Figure S8: (A) Capacitance and (B) ESR of ETE-S and ETE-S/GDH-FAD root supercapacitors as extracted from galvanostatic charge/discharge experiments with and without 100 mM Glucose. (Mean values with standard error)

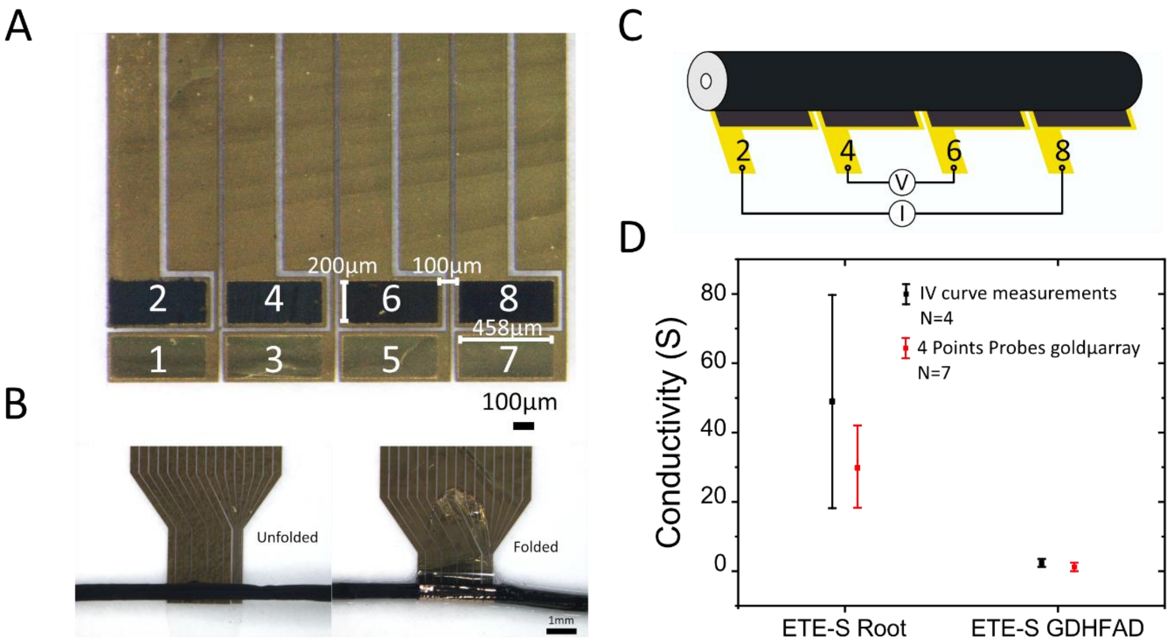

Figure S9: 4-point probe measurements using a conformable gold multielectrode array (MEA) on parylene-c. A) Bright field image of the MEA. The electrodes are numbered from 1 to 8 and their dimensions are reported on the picture with white bars. Electrodes 2,4,6, and 8 are functionalized with electropolymerized ETE-S while electrodes 1,3,5, and 7 are bare gold. B) Photograph of the MEA conformation to a root functionalized with p(ETE-S). Photograph of the unfolded device (left), and photograph of the device folded on the root (right). C) Schematic of the 4-point probe (4PP) measurement. The p(ETE-S) root is placed on the top of the array pre-treated with p(ETE-S). Current is applied between external contacts (2 and 8) while the potential is recorded between internal contacts (4 and 6). D) Conductivity of p(ETE-S) roots p(ETE-S)/GDH-FAD roots as defined with 4PP with the MEA (red boxes, SD showed for N=7) and 2PP IV-TLM measurements with conventional probes (black boxes, SD showed for N=4).

The conductivity measured with the conformable multi electrode array (MEA) in 4-point probe configuration is lower for p(ETE-S)/GDH-FAD) roots in comparison with p(ETE-S) roots. The 4PP conductivity values are compared with two-point probe (2PP) IV-TLM measurements using convectional metallic probes. We observed less variability in conductivity measurements with the MEA- 4PP- than the TLM- 2PP- method that could be due to the better interface of the conformal probe with the root and due to the fact that the interelectrode distance in the MEA is much smaller than in the case of conventional probes.

Methods fr GC-MS qantification

Glucose and sucrose quantification by GC-MS was performed at the Swedish Metabolomics Center in Umeå, Sweden. Information about reagents, solvents, standards, reference and tuning standards, and stable isotopes internal standards can be found as Supplementary information.

Materials: Methanol, HPLC-grade was obtained from Fischer Scientific (Waltham, MA, USA),H20, Milli Q. Regents: Methoxy amine was purchased from Sigma (St. Louis, MO, USA), Pyridine, was purchased from Thermo Fisher Scientific (Waltham, MA, USA) and MSTFA, 1%TMCS, was purchased from Restek (Bellefonte, PA, USA). Standard: Glucose and sucrose were obtained

from Sigma (St. Louis, MO, USA). Internal standards: D-glucose-13C6 and D-sucrose-13C12 were obtained from Sigma (St. Louis, MO, USA).

Sample Preparation: Sample preparation of 500 µl was performed according to Gullberg et al (Gullberg et al. 2004). In detail, 350 µL of extraction buffer (90/10 v/v methanol: water) including internal standards (D-glucose-13C6, D-sucrose-13C12). The sample was shaken with one tungsten bead in a mixer mill at 30 Hz for 2 minutes, the bead was removed, and proteins were precipitated at -20 °C for 1 hour. The sample was centrifuged at +4 °C, 14 000 rpm (18 620g), for 10 minutes. 270 µl of the supernatant was transferred to micro vials and evaporated to dryness in a speed-vac concentrator. Solvents were evaporated to dryness using a nitrogen concentrator and the samples were stored at -80 °C. The samples were analyzed according to a randomized run order.

Calibration curve: A standard curve was prepared (diluted in water) from glucose and sucrose at 17 levels from 0.6 pg/µl to 20 ng/µl). Each level also included the internal standards at fixed concentration of 7.5 ng/µl.

GC-MS Analysis: Derivatization and GC-MS analysis were performed as described previously (Gullberg et al. 2004). 1 µL of the derivatized sample was injected in splitless mode by a L-PAL3 autosampler (CTC Analytics AG, Switzerland) into an Agilent 7890B gas chromatograph equipped with a 10 m x 0.18 mm fused silica capillary column with a chemically bonded 0.18 µm Rxi-5 Sil MS stationary phase (Restek Corporation, U.S.) The injector temperature was 270 °C, the purge flow rate was 20 mL min-1 and the purge was turned on after 60 seconds. The gas flow rate through the column was 1 mL min-1, the column temperature was held at 70 °C for 2 minutes, then increased by 40 °C min-1 to 320 °C, and held there for 2 minutes. The column effluent was introduced into the ion source of a Pegasus BT time-of-flight mass spectrometer, GC/TOFMS (Leco Corp., St Joseph, MI, USA). The transfer line and the ion source temperatures were 250 °C and 200 °C, respectively. Ions were generated by a 70 eV electron beam at an ionization current of 2.0 mA, and 30 spectra s-1 were recorded in the mass range m/z 50 - 800. The acceleration voltage was turned on after a solvent delay of 150 seconds. The detector voltage was 1800-2300 V.

Data Analysis: All non-processed MS-files from the metabolic analysis were exported from the ChromaTOF software in NetCDF format to MATLAB R2021a (Mathworks, Natick, MA, USA), where all data pre-treatment procedures, such as base-line correction, chromatogram alignment, data compression and Multivariate Curve Resolution were performed. Each compound was identified by comparisons of their retention index and mass spectra with libraries of retention time indices and mass spectra. The amount of glucose and sucrose was calculated based on calibration curves.

References:

1. Gullberg J, Jonsson P, Nordström A, Sjöström M & Moritz T. Design of experiments: an efficient strategy to identify factors influencing extraction and derivatization of Arabidopsis thaliana samples in metabolomic studies with gas chromatography/mass spectrometry. Anal Biochem 2004 331 283-295.

2. Schauer N, Steinhauser D, Strelkov S, Schomburg D, Allison G, Moritz T, Lundgren K, Roessner-Tunali U, Forbes MG, Willmitzer L, Fernie AR & Kopka J. GC-MS libraries for the rapid identification of metabolites in complex biological samples. FEBS Lett 2005 579 1332-1337.
